# Single cell, Label free Characterisation of Human Mesenchymal Stromal cell Stemness and Future Growth Potential by Autofluorescence Multispectral Imaging

**DOI:** 10.1007/s12015-024-10778-4

**Published:** 2024-08-27

**Authors:** Jared M. Campbell, Abbas Habibalahi, Adnan Agha, Shannon Handley, Aline Knab, Xiaohu Xu, Akanksha Bhargava, Zhilin Lei, Max Mackevicius, Yuan Tian, Saabah B. Mahbub, Ayad G. Anwer, Stan Gronthos, Sharon Paton, Shane T. Grey, Lindsay Wu, Robert B. Gilchrist, Ewa M. Goldys

**Affiliations:** 1 Graduate School of Biomedical Engineering, University of New South Wales, Sydney, NSW 2052 Australia; 2 Mesenchymal Stem Cell Laboratory, School of Biomedicine, Faculty of Health and Medical Sciences, University of Adelaide, Adelaide, South Australia 5000 Australia; 3 South Australian Health and Medical Research Institute, Adelaide, South Australia 5000 Australia; 4 Garvan Institute of Medical Research, Darlinghurst, New South Wales Australia; 5 Faculty of Medicine, University of New South Wales, Sydney, New South Wales Australia; 6 School of Biomedical Sciences, UNSW Sydney, Sydney, NSW Australia; 7 School of Clinical Medicine, UNSW Sydney, Sydney, NSW Australia

**Keywords:** Mesenchymal stem cell, Autofluorescence, Senescence, Microscopy, Cytometry

## Abstract

**Aim:**

To use autofluorescence multispectral imaging (AFMI) to develop a non-invasive assay for the in-depth characterisation of human bone marrow derived mesenchymal stromal cells (hBM-MSCs).

**Methods:**

hBM-MSCs were imaged by AFMI on gridded dishes, stained for endpoints of interest (STRO-1 positivity, alkaline phosphatase, beta galactosidase, DNA content) then relocated and results correlated. Intensity, texture and morphological features were used to characterise the colour distribution of regions of interest, and canonical discriminant analysis was used to separate groups. Additionally, hBM-MSC lines were cultured to arrest, with AFMI images taken after each passage to investigate whether an assay could be developed for growth potential.

**Results:**

STRO-1 positivity could be predicted with a receiver operator characteristic area under the curve (AUC) of 0.67. For spontaneous differentiation this was 0.66, for entry to the cell-cycle it was 0.77 and for senescence it was 0.77. Growth potential (population doublings remaining) was estimated with an RMSPE = 2.296. The Mean Absolute Error of the final prediction model indicated that growth potential could be predicted with an error of ± 1.86 doublings remaining.

**Conclusions:**

This non-invasive methodology enabled the in-depth characterisation of hBM-MSCs from a single assay. This approach is advantageous for clinical applications as well as research and stands out for the characterisation of both present status as well as future behaviour. The use of data from five MSC lines with heterogenous AFMI profiles supports potential generalisability.

**Graphical Abstract:**

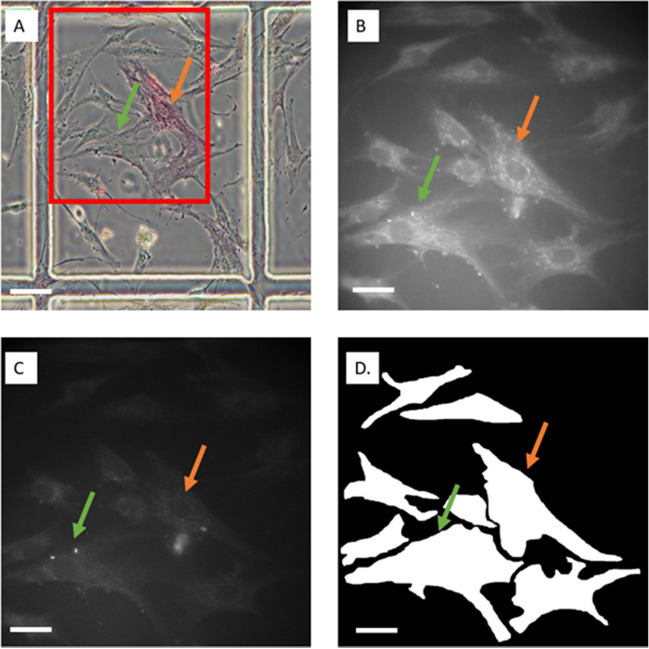

**Supplementary Information:**

The online version contains supplementary material available at 10.1007/s12015-024-10778-4.

## Introduction

Mesenchymal stem or stromal cells (MSCs) have multilineage differentiation potential and immunomodulatory properties – interacting with lymphocytes to achieve immunosuppressive and anti-inflammatory effects [1, 2]. As a result, they have received considerable research interest due to their potential to contribute to knowledge of developmental biology as well as translational applications, including transplantation and regenerative medicine [3].

However, treatment outcomes have proven inconsistent [4], an effect commonly attributed to heterogeneity in the phenotypes of transplanted cells arising from donor variations – e.g. age, sex, disease state [4–6] – and the impact of in-vitro expansion [7]. Prolonged culture, necessary for sufficient cell numbers for therapies, even undertaken according to Good Manufacturing Practice (GMP) [8] increases senescence [9], alters metabolism [7], causes a loss of pluripotency and cellular hypertrophy [10] and has been linked to a lack of immunosuppressive potential and lower survival in transplant recipients [11]. Pre-transplant characterisation of hBM-MSCs for cellular biomarkers of ‘quality’ beyond the canonical characteristics held to define MSC identity have been proposed, including global gene expression [12], miRNA expression [13] or proteome analysis [14]. However, these are labour and time intensive and require a large number of cells making them poor candidates for translation to support clinical interventions [15].

Moreover, it is becoming apparent that conventional assessment of the pooled results of homogenised samples give suboptimal indications of cell lines’ actual characteristics for research and use [16, 17]. Single cell assays are being increasingly utilised to gain precise characterisation of cell line populations, revealing that preparations considered to be functionally homogenous contain meaningful subpopulations with implications for their study and use [11, 16, 18].

Autofluorescence multispectral imaging (AFMI) takes advantage of the presence of endogenous fluorophores – such as nicotinamide adenine dinucleotide (phosphate) (NAD(P)H; excitation maxima 290 and 351 nm, emission maxima 440 and 460 nm)) and flavin adenine dinucleotide (FAD; excitation maxima 450 nm, emission maxima 535 nm)). These metabolic co-enzymes are the principal electron donors and acceptors of oxidative phosphorylation and, along with numerous other autofluorophores with their own biological contributions (e.g. collagen, porphyrins, cytochrome-c) can be used to construct spectral signatures for the prediction and diagnosis of cell and disease states [19]. AFMI works by exposing the biological material of interest to multiple excitation wavelengths and imaging factorial or select combinations of emission wavelengths at the same position and focus to build a “data cube” where each pixel represents the same position in space, but is the product of these different excitation/emission wavelengths [20, 21]. This gives a rich data set, directly reflective of the biological status of the cell, with greater depth than traditional methodologies which target specific autofluorophores as biomarkers. AFMI has been successfully used for the detection of macular degeneration [22], visualisation of radiofrequency ablation lesions e treatment of atrial fibrillation [23], multiple sclerosis characterisation [24]a tumour margin detection across numerous oncological contexts [25]. Furthermore, in human bone marrow MSCs (hBM-MSCs) specifically, AFMI has been shown sensitivity to cellular age [7], position in the cell cycle [20] and levels of reactive oxygen species (ROS) [26].

Importantly, AFMI is non-invasive as it does not require staining, transformation or fixation – which allows studied cells to be subjected to further biological characterisation, including relocation or longitudinal follow up on the literal cells (or daughters) previously assessed – and gives outputs at the single cell level. As such, it has the potential to make a great contribution to the characterisation of MSC quality for therapeutic and research uses, as well as the investigation of cell line heterogeneity [20]. One current use of AFMI in biological applications is through correlative microscopy, where the results from AFMI are correlated with results of known assays and/or pathological presentation, such as correlating the levels of ROS measured by CellROX in cells to autofluorescence images [27] and comparing autofluorescence images of eyes with drusen deposits to the brightfield images [28]. This approach enables the establishment of spectral signatures of cells and tissues, which can be used for longitudinal assessment in follow-up experimental procedures. More recently, AFMI is seeing increased applications in tissue imaging, including differentiating healthy and cancerous tissue and to grade cancer tissue [25]. In this study we aimed to use correlative microscopy with AFMI to develop algorithmic classifiers to categorise single hBM-MSCs by senescent status (beta-galactosidase positivity), spontaneous differentiation (alkaline phosphatase positivity), stemness (STRO-1 positivity), growth velocity (entry to the cell cycle estimated by DNA content) and growth potential (population doublings to full growth arrest).

## Methods

### Cell Culture

hBM-MSC lines, ND0094, HE18K00DB, ND0142, ND0338 and ND0106, were from the posterior iliac crest of informed, consenting donors aged 18–35 years according to the approved procedures (940911a) of the Human Ethics Committee of the Royal Adelaide Hospital, South Australia. STRO-1 + cells were purified by immunomagnetic selection as described previously [29, 30]. Approval for the current project was given by the University of New South Wales low-risk Human Ethics Committee (HC180219). Cell culture was carried out in a humidified incubator at 37^o^C, 5%CO_2_ in α-MEM with sodium bicarbonate, without L-glutamine, ribonucleosides and deoxyribonucleosides (M4526, Sigma) supplemented with Penicillin-Streptomycin (Sigma, P4333), 10% fetal bovine serum (SH30084, Hyclone), L-Glutamine (Gibco, 25030081), L-Ascorbic acid (Sigma, A8960) and sodium pyruvate (Sigma, S8636). Media was refreshed every 3–4 days and cells were subcultured at 80% confluence as described in [21]. Cells (3.75 × 10^5^ cells) were reseeded in 75cm^2^ culture flasks or 35 mm polymer gridded coverslip imaging dishes (0.5 × 10^5^ cells, Ibidi, 81166) for the relocation of cells in correlative microscopy.

### Autofluorescence Multispectral Imaging

Cells were imaged on a warm stage in Hanks Balanced Salt Solution (HBSS), which is fluorescently neutral. AFMI was carried out using an Olympus IX83 microscope and a NuVu electron multiplying charge coupling device (EMCCD, hnu1024) camera. A total of 47 spectral channels were generated using LEDs which fluoresced at excitation wavelengths 358, 371, 377, 381, 385, 391, 397, 400, 403, 406, 412, 418, 430, 437, 451, 457, 469, and 476 ± 5 nm in combination with the collection of emissions using 414 ± 23 nm, 451 ± 53 nm, 575 ± 29.5 nm, 594 long-pass, and 675 ± 33.5 nm filters and a 40× oil objective lens (UAPON340, Olympus). Following hyperspectral autofluorescence imaging, a brightfield image was also obtained using 0.01s exposure for the same field of view. Total imaging time was ~ 4 min and followed the acquisition parameters described in Supplementary material 1. Regions of interest were manually segmented to define individual cells based on the brightfield image. Image preprocessing included cosmic ray removal and applying automatic hyperspectral image restoration using sparse and low-rank modelling (HyRes) [31] for denoising and smoothing. Images were also background subtracted, flattened and calibrated [32] by taking additional water and calibration fluid files. The calibration fluid of pure reference fluorophores (NADH and riboflavin) was taken using a Fluoromax-4 spectrofluorometer, using the same excitation and emission wavelengths used for autofluorescence imaging. The water and calibration files were smoothed using a Cohen-Daubechies-Feauveau wavelet. Data extraction was carried out using the Software for Multidimensional Analysis and Classification (SMIAL) tool https://github.com/EwaGoldys/SMIAL.git .

### Assays

During AFMI the reference grid on the coverslip dishes were used to keep track of the locations of the cells that were imaged. Subsequently the relevant assays were applied to the cells in the dishes (as detailed below) and the grids were used to relocate the imaged fields. Comparisons of the two images than enabled the AFMI images of cells to be associated with the characteristics they were shown to have by the assays. Assessment or fixation was carried out immediately following the completion of AFMI to minimise the impact of cell motility and division. Staining for beta galactosidase (see supplementary Fig. 2 for representative image) and alkaline phosphatase (see supplementary Fig. 3 for representative image) to detect senescent and differentiated cells was carried out with the Senescence β-Galactosidase Staining Kit (Cell Signalling Tech. #9860) and Alkaline Phosphatase Detection Kit (Merck, SCR004), respectively. For immunofluorescence, cells were fixed in 4% formalin for 15 min at room temperature then washed twice in DPBS followed by permeabilization in 0.1% Triton-X for 30 min at room temperature and blocking in 1% bovine serum albumin for 30 min at room temperature. hBM-MSCs were then stained overnight at 4^o^C with 1:400 anti-STRO-1 mouse monoclonal antibody (39-8401, ThermoFisher) in 10% normal goat serum (1620-064, Gibco). Cells were washed twice with DPBS then stained with goat anti-mouse Alexa Fluor 594 (A-11005, Invitrogen), washed twice more and stained with DAPI (R37606, Invitrogen). After a final wash, cells were imaged on an Olympus FV3000 confocal microscope with a fully open pinhole to capture total fluorescence (see supplementary Fig. 4 for representative image). Fluorescence intensity data was extracted using the graphics software ImageJ, normalised against background and cell area then classified based on the assessment of positivity/negativity (STRO-1) or plotted on a histogram to indicate position in the cell cycle based on total DAPI intensity [33] (supplementary Fig. 5).

### Spectral Modelling

Intensity, morphological and textural features were generated for each cell using Matlab 2023b (see Supplementary material 2 for complete listings). Cells were classified into the relative groupings (e.g. differentiated, senescent) based on the results of the assays described above and a supervised machine learning algorithm was applied to differentiate between the groups using the SMAL GUI machine learning tool. Using a combination of all features, the minimum redundancy maximum relevance (MRMR) method was used to select the top features (number mediated by the size of the individual dataset) before being applied to principal component analysis (PCA) followed by linear discriminant analysis (LDA) for dimensionality reduction. LDA additionally achieves maximal separation of the groups, maximising between-group distance while minimising within-group variance. Where there were class imbalances in the number of cells per group (in the training set), adaptive synthetic (ADASYN) up-sampling [34] or alternatively random down sampling was used on the minority or majority class dependent on the characteristics of the dataset (e.g. size). To differentiate alkaline phosphatase positive MSCs from negative cells an LDA classifier was employed. Differentiation of STRO-1 positive MSCs from negative, as well as classification of senescent and non-senescent cells was performed using a linear SVM classifier. A polynomial support vector machine (SMV) was employed to discriminate between cell cycle phases (G1 vs. G2, S and M phase). All accuracy performance metrics provided are for the test folds. A receiver operating characteristic (ROC) area under the curve (AUC) analysis quantified the accuracy of models. An AUC value of 1.0 would indicate complete accuracy, i.e., all data points correctly classified, and a value of 0.5 would indicate no better odds than chance, i.e., the portion of data points correctly classified is directly proportional to what would be achieved by randomisation. Specificity and sensitivity were also measured. An inclusive illustration of the full image acquisition and modelling strategy is provided in supplementary Fig. 1.

## Results

**Fig. 1:**
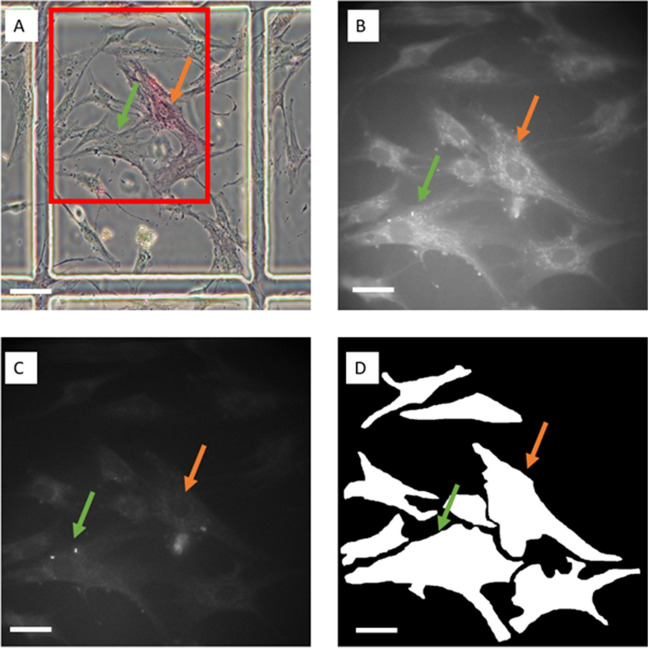
Example correlative microscopy approach undertaken in this study. (**A**) ND0142 image captured by light microscopy after *AFMI*. Alkaline-phosphatase positivity was determined using the Alkaline Phosphatase Detection Kit (Merck, SCR004). Positive cells stained red (orange arrow) and negative cells did not change colour (green arrow). The red box shows the region of interest that the AFMI microscope could capture; (**B**) *AFMI* image of the same sample showing spectral channel 5 (ex/em = 358/451nm); (**C**) *AFMI* of the same sample showing spectral channel 21 (21 ex/em = 457/575m); (**D**) Mask image produced to segment cells for classification. Scale bar for A: 50 μm; scale bar for B-D: 25 μm

AFMI images of hBM-MSCs were obtained with subsequent staining and correlative microscopy for the different characteristics of interest (i.e., STRO-1, senescence, cell division, and spontaneous differentiation (Fig. 1B and C)). Additionally, cumulative population doublings were tracked per passage (along with the collection of AFMI images) as cells were cultured towards full growth arrest in order to retrospectively calculate population doublings remaining. The corresponding results of the biological assays were used to classify the cell images according to their status (e.g. differentiated vs. non-differentiated; Fig. 1). Cells were then masked by manually drawing outlines around the cell borders (Fig. 1D) and processed to classify them into groups based on the characteristic studied. Figure 1A is a light-microscopy image of the alkaline phosphatase stain that was performed after AFMI. The red box highlights the field of view of that the AFMI captured. Figure 1B and C show sample AFMI images from the alkaline phosphatase experiment. The orange arrow represents an alkaline positive cell, which was classified as a cell undergoing spontaneous differentiation. The green arrow represents an alkaline phosphatase negative cell, which was classified as a cell not undergoing spontaneous differentiation. Five lines from different donors were used to demonstrate that these models could be generated based on generalisable characteristic rather than being line specific. Based on Kruskal–Wallis test of mean channel intensities, 43% of channels had significant differences between lines (supplementary material 4), as such the findings described below should be considered in the context of meaningful heterogeneity.

**Fig. 2:**
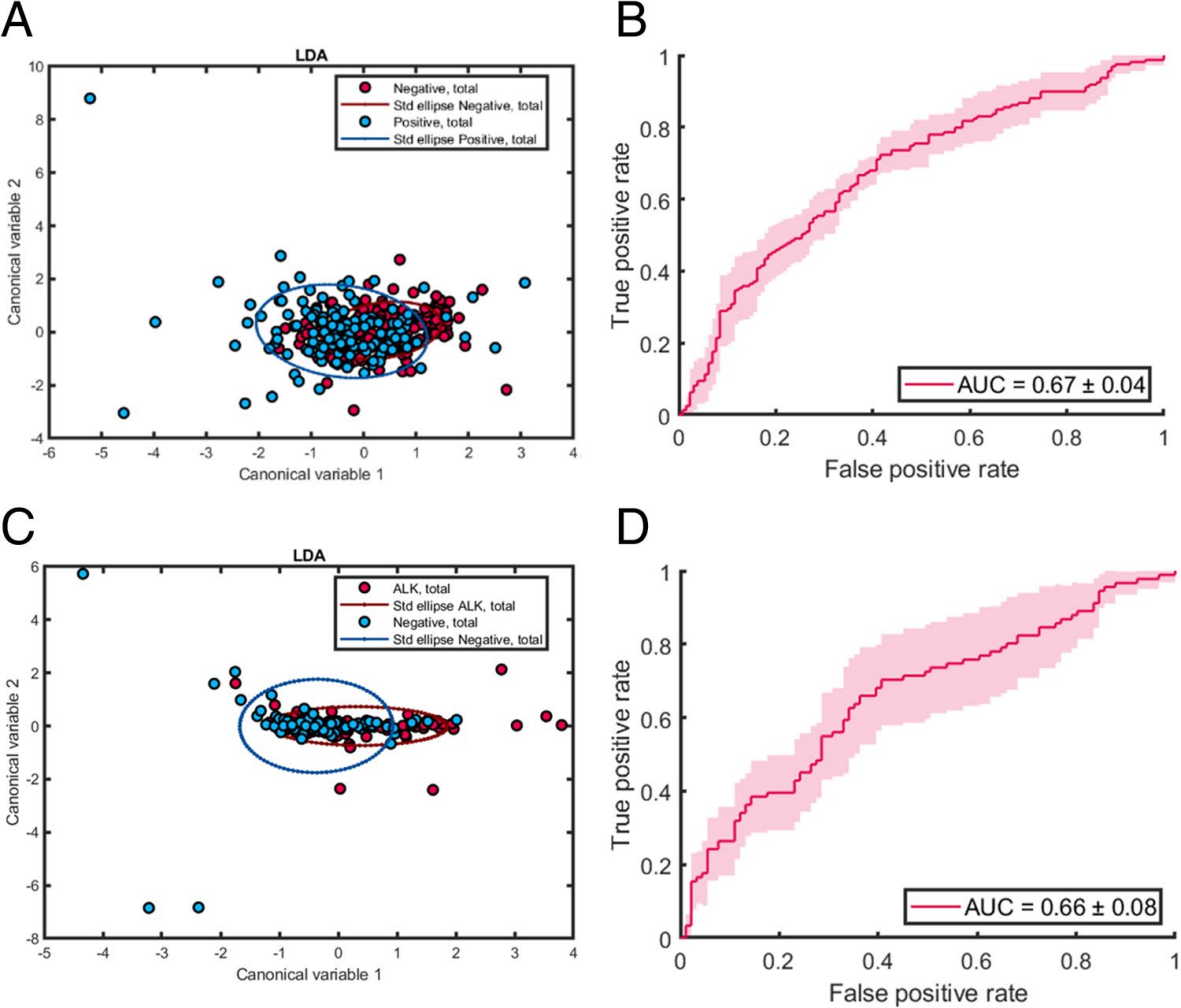
Assessment of MSC status by *AFMI* microscopy of autofluorescence. For LDA plots, corresponding 95% confidence intervals are drawn around the mean to illustrate separation. ROC curves have been drawn with corresponding error values (pink regions) based on the cross validation. (**A**) STRO-1 positive MSCs were discriminated from negative, final model illustrated by LDA scatter plot (based on total data set 289, > 5 technical replicates). (**B**) STRO-1 ROC curve (based on testing data set 29) using a linear SVM classifier. (**C**) Differentiated (alkaline phosphatase positive MSCs) were discriminated from negative cells final model illustrated by LDA scatter plot (based on total data set 182 3 technical replicates); (**D**) alkaline phosphatase ROC curve (based on training data set 18) using an LDA classifier

Some discrimination could be achieved for STRO-1 (AUC = 0.68, Fig. 2A and B) with high sensitivity (0.86 + 0.10) but low specificity (0.3 + 0.16). Similarly for alkaline phosphatase (spontaneous differentiation), an AUC of 0.66 + 0.08 (Fig. 2C and D) was achieved, but with an associated sensitivity of 0.55 + 0.20 and specificity 0.73 + 0.11.

**Fig. 3:**
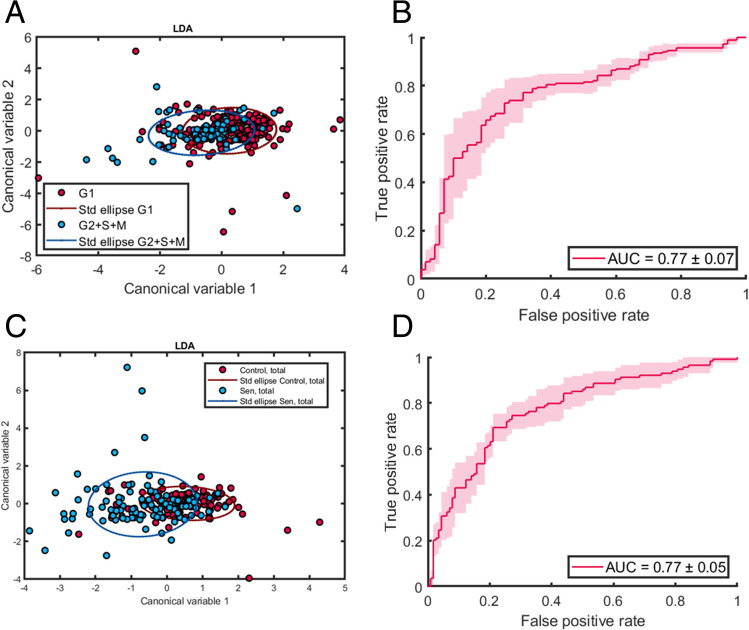
Assessment of MSC status by *AFMI* microscopy of autofluorescence. For LDA plots, corresponding 95% confidence intervals are drawn around the mean to illustrate separation. ROC curves have been drawn with corresponding error values (pink regions) based on the cross validation. (**A**) Discrimination of non-dividing MSCs (in the G1 phase of the cell cycle) from dividing MSCs (in the G2, S or M phases of the cell cycle) final model illustrated by LDA scatter plot (based on total data set, 254, > 5 technical replicates). (**B**) cell division ROC curve (based on testing data set 51) using a polynomial SVM classifier. (**C**) Senescent (beta galactosidase positive cells) were discriminated from non-senescent cells, final model illustrated by LDA scatter plot (based on total data set 268, 3 technical replicates); (**D**) beta galactosidase ROC curve (based on training data set 54) using a linear SVM classifier

Senescent and non-senescent cells could be discriminated with an AUC = 0.87 (Fig. 3A), demonstrating that AFMI was able to identify cell with no remaining replicative lifespan.

Better discrimination was achieved for properties associated with cell growth (Fig. 3) with an AUC of 0.77 ± 0.07 for discriminating quiescent cells (G1 phase) from those which had entered the cell cycle (Fig. 3A and B, S, G2 or M phase). The associated sensitivity and specificity values were 0.68 ± 0.13 and 0.74 ± 0.12, respectively. Less accuracy was achieved for differentiating stages of the cell cycle more granularly, with the differentiation of cells in the process of DNA replication (S phase) from cells which had completed replication (G2 and M phase) having an AUC of 0.68 ± 0.12. Differentiation of M phase cells was not attempted as there were insufficient data points for modelling (2). Senescent cells (those which are incapable of undergoing further cell division, indicated by positive beta galactosidase staining) were also discriminated with an AUC of 0.77 (Fig. 3. C and D), while sensitivity was 0.76 ± 0.11 and specificity was 0.67 ± 0.19.

These findings demonstrated that AFMI was able to identify cell with no remaining replicative lifespan. As such, we investigated whether AFMI could be used to estimate cells’ number of remaining divisions in general. Correlative microscopy was not used for this experiment as there is no ‘gold-standard’ test for predicting remaining divisions in a given cell’s lifespan. Instead, AFMI was applied to hBM-MSCs at each passage from passage 2 through to growth arrest. Total population was calculated at each subculture to estimate cumulative population doublings undergone at each round of imaging, which could then be retrospectively used to track back how many rounds of population doublings those cells had remaining after the end value had been determined on the completion of culture, i.e., full growth arrest. As the different hBM-MSC lines were variable in how many population doublings they ultimately achieved, this value does not directly equivalate with passage number within the overall population.

To predict doublings remaining from the hyperspectral data, a multidimensional feature array was created, where rows were individual cells and the columns represented the various spectral intensity (and morphological) features extracted from the AFMI Data (Supplementary Material 2). After feature extraction, highly correlated variables were identified and removed and the dataset normalised by z-scoring before applying a filter based feature selection to select the top 100 features. The filter method used was minimum redundancy maximum relevance [35] which uses the provided labels to select features which are maximally relevant for class prediction and minimally redundant. The dimensionally reduced dataset was then used to inform a Linear Discriminant Embedding [36]. Class labels were created from cell doubling values remaining rounded to the nearest whole number giving 24 values ranging from 28 to 0 doublings remaining (supplementary Fig. 6). Due to variation in the growth dynamics of the different lines not every value included data from every line – particularly the higher values which some lines did not reach. To test the prediction of doublings remaining, a test set consisting of half a doubling sample (randomly selected) was withheld from the LD training and subsequently projected onto the trained embedding. The doublings remaining was predicted for each test point by using a coarse k-nearest neighbours (KNN) [37, 38] approach, where the distances to the 400 nearest points were calculated and the label of the set of points from a single label with the closest average distance was given to the test point. This same process was performed for each of the 24 doubling values.

**Fig. 4:**
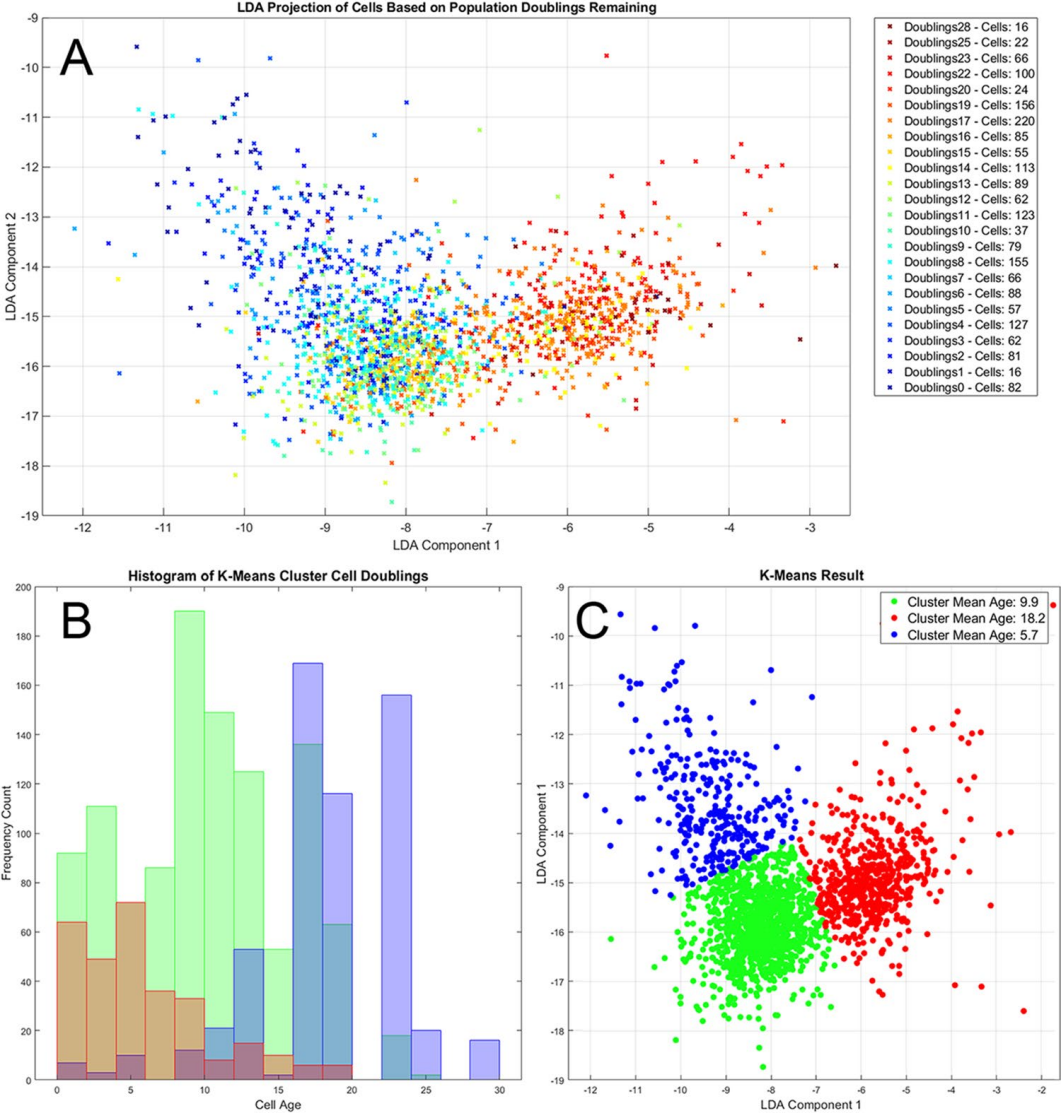
A) Plot of cells coloured by population doublings remaining projected onto the first two Linear Discriminant components, shows a progression from young cells (Red) on the right, to old cells (Blue) on the left. B**)** and C**)** Unsupervised k-means clustering on the LDA components identifies 3 main age clusters with the mean age identified for each cluster and the exact age distribution identified in the histogram. The red and blue clusters are predominantly young or old cells respectively, the green cluster centres on the middle age cells however still contains more young cells than the entire red group

Projection of the top two components of the Linear Discriminant (Fig. 4A) showed that the first component effectively separated cells according to their doublings remaining. This indicated a clear progression from young to old that is captured by the hyperspectral features. Applying unsupervised K-means clustering (Fig. 4C) on the LD components consistently identified 3 main clusters: A young cluster (Red), a middle aged cluster (Green) and an old age cluster (Blue). As shown by the cluster histogram of cell age frequencies, the red and blue group are predominantly young or old cells respectively while the green group had a large spread of cells from various population doublings with a peak at 9–11 doublings remaining. This is likely caused by the fact that population doublings remaining were recorded for a whole passage, however the individual cells within would vary in their actual age state and growth potential, which would then be reflected in their hyperspectral characteristics.

**Fig. 5:**
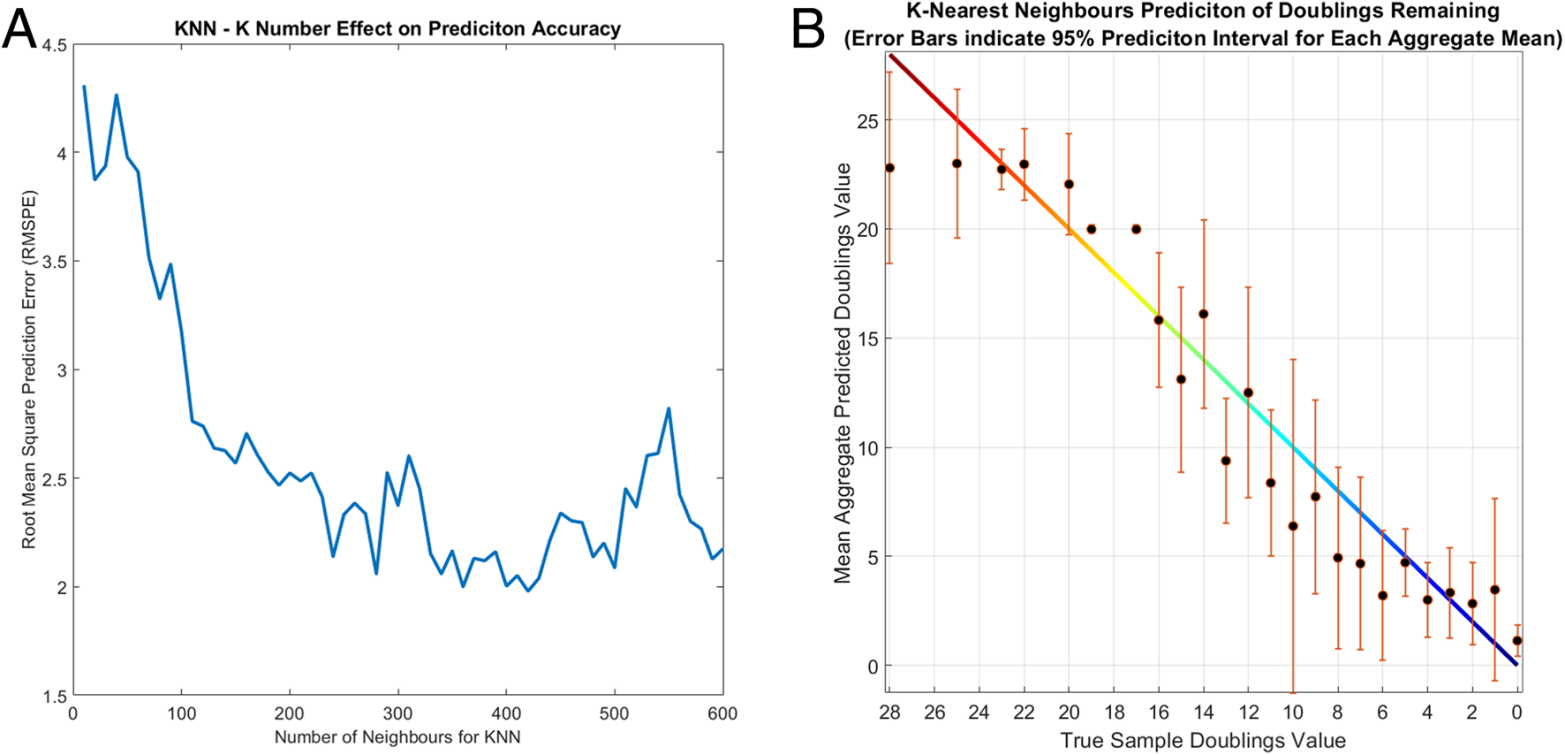
(A) Effect of number of neighbours used for KNN prediction on the overall Root Mean Square Error (RMSE). (B) KNN prediction of test samples based on the mean aggregate point score. (RMSPE = 2.296). Error bars represent the 95% prediction interval. i.e. there is a 95% probability that that the true value lies within the error bars

Due to this within-passage cellular heterogeneity it is not appropriate to infer the likely population doublings remaining for a line based on the characteristics of a single cell. As such, for each test sample, an aggregate score was assigned based on the median of the individual cell label predictions. The aggregate score for a doubling group test sample was predicted 30 times, randomising the test sample points each time. This was done to check the effect of the test point selection on the final aggregate prediction. The mean aggregate scores could then be plotted against the true values to assess how accurate the aggregate score could be in reflecting the true population doublings remaining (Fig. 5).

The prediction of aggregate sample age suggested that the full range of embedding dimensions used to inform the KNN classifier were necessary to properly separate the doubling groups that are clustered within the first two dimensions especially within the middle age cells. The use of such a coarse KNN (k = 400) to provide the best results for individual point prediction is most likely due to the heterogeneity of the cell populations, meaning that a large KNN has a more global view of the point in the embedded space. This balance of local to global view was tested for a range of nearest neighbour values (Fig. 5A) and showed the lowest prediction error at around 400 neighbours. The aggregate prediction scores for each doubling test sample varied between each of the runs, as shown error bars of Fig. 5A. Since a new random sample was used for each aggregate scoring, the larger error bars indicate that point selection for the prediction played a large role in the final aggregate score. The mean of aggregate predictions however do align much better with the true sample doubling value. As each doubling group does not have a consistent number of cells, there is a potential for under-sampling of the true heterogeneity of the cell population at each doubling value which will affect the prediction accuracy. The prediction accuracy for the youngest cells appears to plateau at around 28 to 20 population doublings remaining. This could indicate that there is little difference in the hyperspectral signatures between the youngest cell passages. However, after 22 doublings remaining, as more cells start to show the first detectable signs of aging, the distribution of cell signatures for a doubling population becomes more distinct leading to better predictions. The KNN prediction accuracy was optimised using the Root Mean Square Prediction Error (RMSPE), treating the KNN as a regression-based prediction due the final values almost never exactly matching the true doublings value. The RMSPE was used to optimise the number of neighbours for the KNN (Fig. 5A), where a lower value indicates a higher prediction accuracy with the final model resulting in an RMSPE of 2.296. The Mean Absolute Error of the final prediction model was 1.86 indicating that doublings could be predicted with an error of ± 1.86 doublings remaining. This is however the average error and as shown in Fig. 5B, this error is expected to be larger for the younger passages. With a larger dataset that more completely captures the heterogeneity of each doubling population, the prediction model could be further optimised to reduce the prediction error and gain a better understanding of bounds within which reliably accurate predictions could be made for new sample populations.

## Discussion

In our investigation we achieved limited success in using AFMI to estimate MSC pluripotency status (i.e. STRO-1 and alkaline phosphatase positivity) with an AUC of 0.66 and 0.67, respectively. We were more successful in characterising properties relating to cell growth dynamics, including entry to the cell cycle and the presence of senescence (AUC = 0.77 in both cases, which meets the threshold to be considered “acceptable” accuracy [39]). These findings were made in the context of the pooled data of five MSC lines from different donors. This would be expected to be associated to result in significant heterogeneity (an expectation supported by our observation of significant differences between the AFMI intensity values between the cell lines (supplementary material 4)). This heterogeneity likely resulted in lower accuracy than would have been obtained if we had undertaken to construct models specific to the cell line in which they were trained and tested. However, by demonstrating that the strategy is robust to the presence of inter-patient variability generalisability and implementation are supported.

Additionally, we were able to demonstrate that MSC line future growth potential can be estimated based on AFMI of early passage cells.Estimating cell growth potential is a novel achievement, which will allow for the estimation of remaining “useful cell life,” particularly in aged hBM-MSC cultures. Continuous serial passaging of hBM-MSCs increases the time required for population doubling [7]. Tokumitsu et al. (2009) calculated the generation time of hBM-MSC from three donors as 55 to 107 h [40]. Given this heterogeneity, knowing the number of population doublings remaining at later passages helps prevent subculturing cell populations that do not have a high useful cell life left. This is especially important for regenerative medicine to ensure that hBM-MSCs being cultured are still clinically useful at the time of application.

Limitations of this work include that the spectral signatures modelled for the various characteristics of interest are themselves only indicators of the assays investigated, e.g. alkaline phosphatase positivity and beta galactosidase positivity, which are good markers of spontaneous differentiation and senescence, are not essential and exclusive elements of these statuses. Moreover, there is some delay between the initiation of AFMI and fixation, during which cell status’ could change (this is especially true for cell cycle modelling). The use of a large number of data points in the training sets should mitigate the impact of this issue, but it could still impact the accuracy of our algorithms. All imaged cells were included in the data set where their identity and characteristics could be reliably identified. However, this approach will still have had a bias against large hypertrophic cells (such as those which appear with increasing frequency as MSCs age) due to a smaller number of them fitting within a given field of view compared to smaller cells, and the increased likelihood that they will be cut off by the edge of the image. Additionally, the imaging protocol applied was relatively long (~ 4 min) which would have implications ease of implementation in an applied environment. However, as only informative channels included in the final models would need to be retained this would be considerably reduced.

Consideration must also be given to AFMI’s performance relative to the basic assessment of morphological features relating to cell size and shape. These features were made available to the model (Supplementary material 2), but were rarely included (only four times across all models, not more than twice in a single model (senescence); supplementary material 3). For prediction of entry into cell cycle, a pilot assessment was performed where only morphological features were made available, this resulted in an AUC of 0.54, indicating almost no predictive potential.

The ability to estimate multiple clinically relevant hBM-MSC parameters from a single, non-invasive assay would improve the clinical application of these cells by reducing the time, resources and cell material needed for their characterisation. Our approach for estimating population doublings remaining stands out for not simply characterising the status of cells, but predicting their future outcomes and growth potential. Additionally, as AFMI gives output at the single cell level without destroying the material being studied it has the potential to advance stem cell research by enabling the precise study of the impact of exposures on individual cells’ characteristics as well as longitudinal evaluation. In particular the combination of AFMI with cell picking technologies such as ‘laser tweezers’ could allow the selection of cells with particular characteristics for study without the potentially confounding impact of staining or transformation with reporter genes. Furthermore, its non-invasive nature means that other, destructive assays could be applied to cells and correlated to the outcomes of the AFMI (e.g. after AFMI analysis cells could be fixed and assessed for the presence of a particular protein, whose expression could then be correlated to senescence, differentiation and cell cycle status on a cell-by-cell basis). As such, these results show that AFMI has great potential to accelerate both hBM-MSC therapeutics and research. 

## Supplementary Information

Below is the link to the electronic supplementary material.

Supplementary file1 (DOCX 1.34 MB)

## Data Availability

Data will be made available to qualified researchers on reasonable request.
