# Supplementary material for: Single cell, Label free Characterisation of Human Mesenchymal Stromal cell Stemness and Future Growth Potential by Autofluorescence Multispectral Imaging
Source: Stem Cell Rev Rep. 2024 Aug 27;20(8):2283–92. doi: 10.1007/s12015-024-10778-4 (PMC11554749; doi:10.1007/s12015-024-10778-4)

**Supplementary Materials**

**Supplementary material 1: Imaging protocol**

| **Channel** | **Excitation (nm) ± 5nm** | **Emission (nm)** | **Exposure time (s)** | **EM Gain** | **Averages** |
| --- | --- | --- | --- | --- | --- |
| 1 | '358' | '414' ±23 | '2' | '300' | '3' |
| 2 | '371' | '414' ±23 | '2' | '300' | '3' |
| 3 | '377' | '414' ±23 | '2' | '300' | '3' |
| 4 | '381' | '414' ±23 | '2' | '300' | '3' |
| 5 | '358' | '451' ±53 | '2' | '300' | '3' |
| 6 | '371' | '451' ±53 | '2' | '300' | '3' |
| 7 | '377' | '451' ±53 | '2' | '300' | '3' |
| 8 | '381' | '451' ±53 | '2' | '300' | '3' |
| 9 | '358' | '575' ±29.5 | '2' | '300' | '3' |
| 10 | '371' | '575' ±29.5 | '2' | '300' | '3' |
| 11 | '377' | '575' ±29.5 | '2' | '300' | '3' |
| 12 | '381' | '575' ±29.5 | '2' | '300' | '3' |
| 13 | '391' | '575' ±29.5 | '2' | '300' | '3' |
| 14 | '400' | '575' ±29.5 | '2' | '300' | '3' |
| 15 | '403' | '575' ±29.5 | '2' | '300' | '3' |
| 16 | '406' | '575' ±29.5 | '2' | '300' | '3' |
| 17 | '412' | '575' ±29.5 | '2' | '300' | '3' |
| 18 | '418' | '575' ±29.5 | '2' | '300' | '3' |
| 19 | '430' | '575' ±29.5 | '2' | '300' | '3' |
| 20 | '437' | '575' ±29.5 | '2' | '300' | '3' |
| 21 | '457' | '575' ±29.5 | '2' | '300' | '3' |
| 22 | '469' | '575' ±29.5 | '2' | '300' | '3' |
| 23 | '476' | '575' ±29.5 | '2' | '300' | '3' |
| 24 | '358' | '594' | '1.5' | '300' | '2' |
| 25 | '371' | '594' | '1.5' | '300' | '2' |
| 26 | '377' | '594' | '1.5' | '300' | '2' |
| 27 | '381' | '594' | '1.5' | '300' | '2' |
| 28 | '391' | '594' | '1.5' | '300' | '2' |
| 29 | '400' | '594' | '1.5' | '300' | '2' |
| 30 | '406' | '594' | '1.5' | '300' | '2' |
| 31 | '418' | '594' | '1.5' | '300' | '2' |
| 32 | '437' | '594' | '1.5' | '300' | '2' |
| 33 | '457' | '594' | '1.5' | '300' | '2' |
| 34 | '476' | '594' | '1.5' | '300' | '2' |
| 35 | '358' | '675' ±33.5 | '1.5' | '300' | '2' |
| 36 | '371' | '675' ±33.5 | '1.5' | '300' | '2' |
| 37 | '377' | '675' ±33.5 | '1.5' | '300' | '2' |
| 38 | '381' | '675' ±33.5 | '1.5' | '300' | '2' |
| 39 | '391' | '675' ±33.5 | '1.5' | '300' | '2' |
| 40 | '400' | '675' ±33.5 | '1.5' | '300' | '2' |
| 41 | '406' | '675' ±33.5 | '1.5' | '300' | '2' |
| 42 | '412' | '675' ±33.5 | '1.5' | '300' | '2' |
| 43 | '418' | '675' ±33.5 | '1.5' | '300' | '2' |
| 44 | '437' | '675' ±33.5 | '1.5' | '300' | '2' |
| 45 | '457' | '675' ±33.5 | '1.5' | '300' | '2' |
| 46 | '469' | '675' ±33.5 | '1.5' | '300' | '2' |
| 47 | '476' | '675' ±33.5 | '1.5' | '300' | '2' |
| 48 | '' | '575' ±29.5 | '0.01' | '300' | '1' |

**Supplementary material 2: Extracted features**

**Intensity features**

- Mean intensity
- Median intensity
- Standard deviation
- Minimum
- Maximum
- Mode
- Skewness
- Kurtosis
- Variance
- Top 10% pixel values
- 75^th^ percentile pixel values
- 25^th^ percentile pixel values
- 50^th^ percentile pixel values
- Channel ratios: ratio of the mean intensity from two channels
- Channel products: products of the mean intensity from two channels
- Average value of the top 30% pixel values
- Ratio of the average value of the top 10% from one channel and top 40% from a second channel
- Total intensity value: sum of all pixel values in a cell

**Morphology features**

Morphology features were obtained using the brightfield image. See <https://au.mathworks.com/help/images/ref/regionprops.html> for definitions.

- Area
- Perimeter
- Major axis length
- Minor axis length
- Eccentricity
- Orientation
- Convex area
- Circularity
- Filled area
- Euler number
- Equivalent diameter
- Solidity
- Extent
- Maximum ferret diameter
- Maximum ferret angle
- Minimum ferret diameter
- Minimum ferret angle

**Texture**

Haralick texture features were obtained from (https://www.mathworks.com/matlabcentral/fileexchange/58769-haralicktexturefeatures) using a gray-level co-occurrence matrices with four offsets: one pixel to the right, one pixel up, one pixel 45° to the right and one pixel 45° to the left.

- Angular second moment
- Contrast
- Correlation
- Variance
- Homogeneity
- Sum average
- Sum variance
- Sum entropy
- Entropy
- Difference variance
- Difference entropy
- Information measure of correlation I
- Information measure of correlation II
- Maximum correlation coefficient

Supplementary material 3: Included features

**STRO-1**

1. Channel 3* Channel 24
2. Channel 8/ Channel 19
3. Channel 35_Maximum_Correlation_Coefficient
4. Channel 36_Sum_Average
5. Euler_Number
6. Channel 39_Maximum_Intensity
7. 10% Channel 8/40% Channel 6
8. Channel 21/ Channel 14
9. Channel 24_Mode
10. Channel 5_Minimum_Intensity
11. Channel 37_25th_Percentile
12. 10% Channel 22/40% Channel 25

**Cell division**

1. Minimum intensity channel 21
2. Ratio mean intensity channel 38 to mean intensity channel 12
3. Ratio mean intensity channel 5 to mean intensity channel 46
4. Ratio mean intensity channel 36 to mean intensity channel 45
5. Minimum intensity channel 36
6. Median intensity channel 46
7. Ratio of top 10% pixel values channel 27 to top 40% pixel values channel 46
8. Maximum intensity channel 12
9. Ratio of top 10% pixel values channel 4 to top 40% pixel values channel 3
10. Ratio of top 10% pixel values channel 37 to top 40% pixel values channel 35

**Senescence**

1. Channel 42_Maximum_Correlation_Coefficient
2. Channel 10_Skewness
3. Channel 13_Brightest10
4. Channel 20/ Channel 27
5. Channel 10_Minimum_Intensity
6. Channel 36/ Channel 41
7. Channel 45/ Channel 46
8. Channel 22/ Channel 13
9. 10% Channel 26/40% Channel 34
10. Channel 36_Information_Measure_of_Correlation_II
11. Channel 35_Total Intensity
12. 10% Channel 13/40% Channel 9
13. Maximum_Feret_Diameter_unit
14. Minimum feret diameter_unit
15. 10% Channel 13/40% Channel 38

**Alk phos**

1. 10% Channel 23/40% Channel 30
2. Minimum feret angle
3. Channel 25_25th_Percentile
4. 10% Channel 21/40% Channel 32
5. 10% Channel 22/40% Channel 29
6. Channel 30_25th_Percentile
7. 10% Channel 22/40% Channel 28
8. 10% Channel 18/40% Channel 29
9. 10% Channel 23/40% Channel 28
10. 10% Channel 20/40% Channel 29
11. 10% Channel 22/40% Channel 30
12. 10% Channel 23/40% Channel 29
13. 10% Channel 19/40% Channel 29
14. 10% Channel 23/40% Channel 32
15. 10% Channel 22/40% Channel 32

Supplementary material 4: Differences in mean channel intensities across lines, based on Kruskal–Wallis test. p<0.05 highlighted yellow.

| Feature | p |
| --- | --- |
| x1_Mean_Intensity | 0.06573035 |
| x2_Mean_Intensity | 0.000335737 |
| x3_Mean_Intensity | 0.000693539 |
| x4_Mean_Intensity | 0.000593239 |
| x5_Mean_Intensity | 0.002681751 |
| x6_Mean_Intensity | 1.05184E-05 |
| x7_Mean_Intensity | 1.3724E-05 |
| x8_Mean_Intensity | 0.000779453 |
| x9_Mean_Intensity | 0.048492135 |
| x10_Mean_Intensity | 0.052090818 |
| x11_Mean_Intensity | 0.062388071 |
| x12_Mean_Intensity | 0.139419788 |
| x13_Mean_Intensity | 0.569359247 |
| x14_Mean_Intensity | 0.612936124 |
| x15_Mean_Intensity | 0.608330514 |
| x16_Mean_Intensity | 0.693735358 |
| x17_Mean_Intensity | 0.856750534 |
| x18_Mean_Intensity | 0.49580764 |
| x19_Mean_Intensity | 0.765699473 |
| x20_Mean_Intensity | 0.495327978 |
| x21_Mean_Intensity | 0.404496623 |
| x22_Mean_Intensity | 0.421954083 |
| x23_Mean_Intensity | 0.372431987 |
| x24_Mean_Intensity | 0.097354909 |
| x25_Mean_Intensity | 0.003870488 |
| x26_Mean_Intensity | 0.005741934 |
| x27_Mean_Intensity | 0.029998176 |
| x28_Mean_Intensity | 0.077081907 |
| x29_Mean_Intensity | 0.552923234 |
| x30_Mean_Intensity | 0.404762662 |
| x31_Mean_Intensity | 0.239654016 |
| x32_Mean_Intensity | 0.122198309 |
| x33_Mean_Intensity | 0.613050822 |
| x34_Mean_Intensity | 0.73597589 |
| x35_Mean_Intensity | 0.07906332 |
| x36_Mean_Intensity | 0.000224455 |
| x37_Mean_Intensity | 0.001797445 |
| x38_Mean_Intensity | 0.000137771 |
| x39_Mean_Intensity | 0.003288934 |
| x40_Mean_Intensity | 0.380370486 |
| x41_Mean_Intensity | 0.031414062 |
| x42_Mean_Intensity | 0.13505757 |
| x43_Mean_Intensity | 0.002983297 |
| x44_Mean_Intensity | 0.156342275 |
| x45_Mean_Intensity | 0.001871862 |
| x46_Mean_Intensity | 0.015023201 |
| x47_Mean_Intensity | 0.036143839 |

Supplementary Figure 1: Flow diagram of experimental/analytic strategy


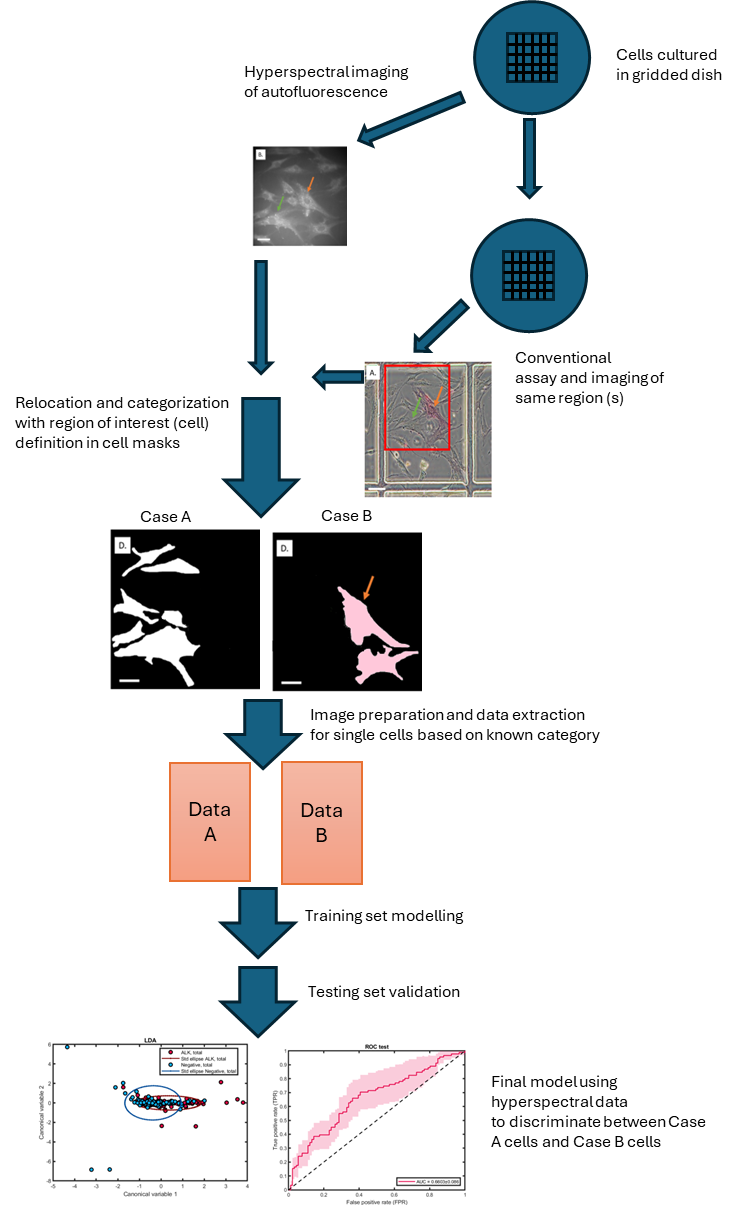


Supplementary Figure 2: Representative image of beta galactosidase positivity. Grid square is 500µm


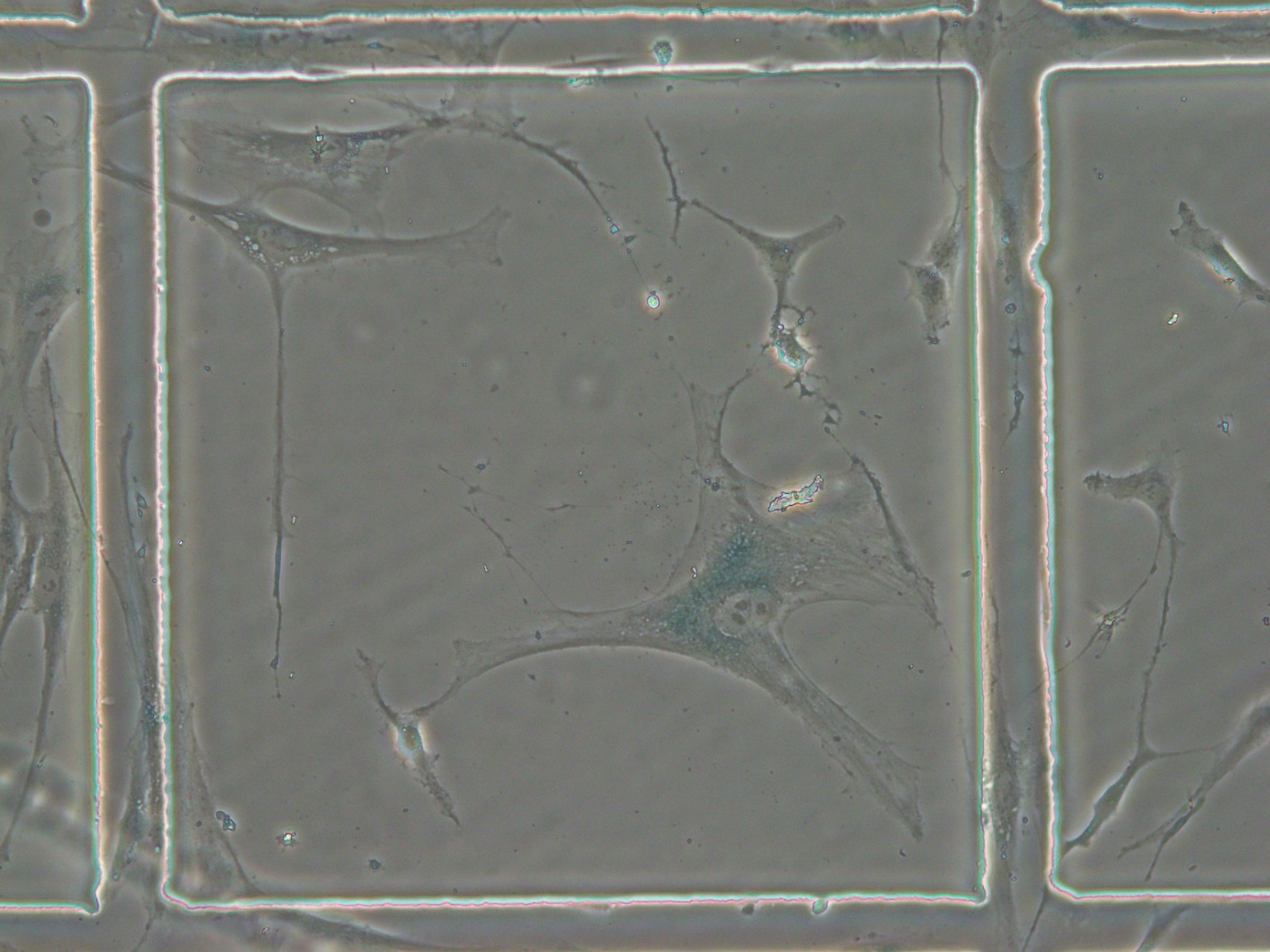


Supplementary Figure 3: Representative image of alkaline phosphatase positivity. Grid square is 500µm


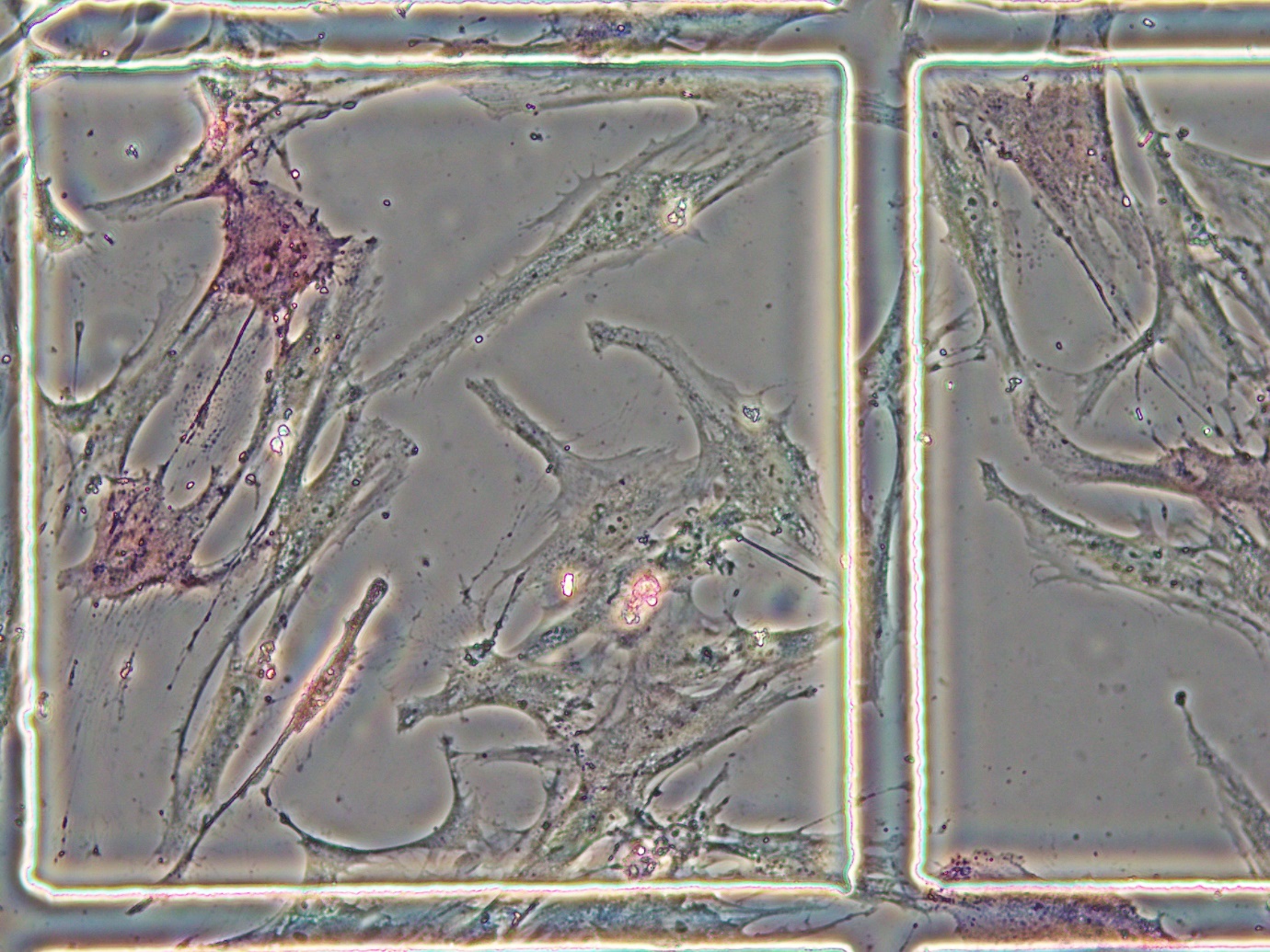


Alkaline phosphatase/ spontaneous differentiation

Grid square is 500µm

Supplementary Figure 4: Representative image of STRO1 positivity. A) STRO-1 staining, B) Nuclear DAPI staining, C) Brightfield image of the same region. Orange arrow indicates a STRO1 positive cell, blue arrow indicates a STRO1 negative cell.


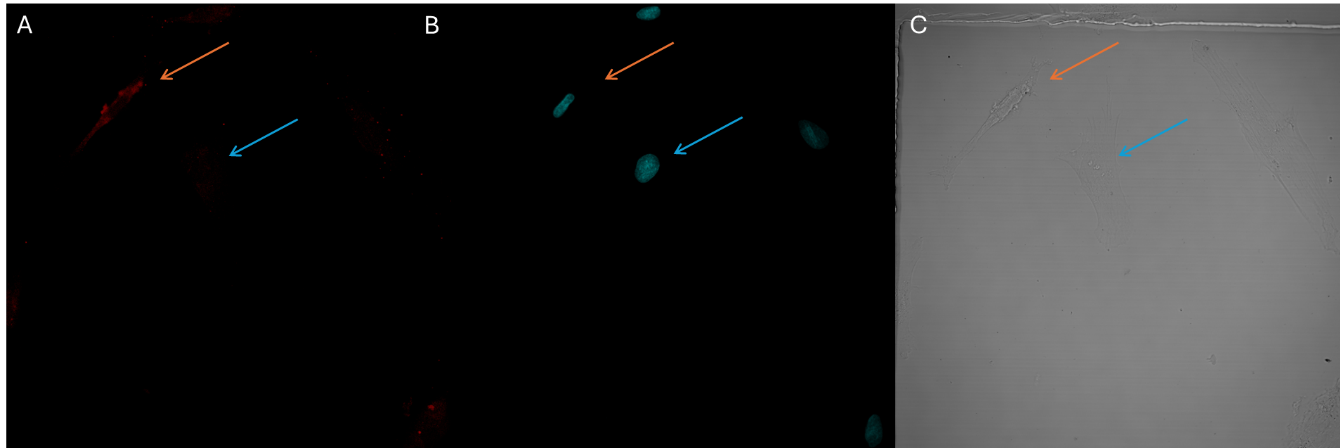


Supplementary Figure 5: Histogram of DAPI nuclear intensity. Cells were classified as G1 if <81242, S phase if 8142-121242, and G2 if >121242. M phase was judged morphologically by the presence of a visibly condensed or dividing nucleus.


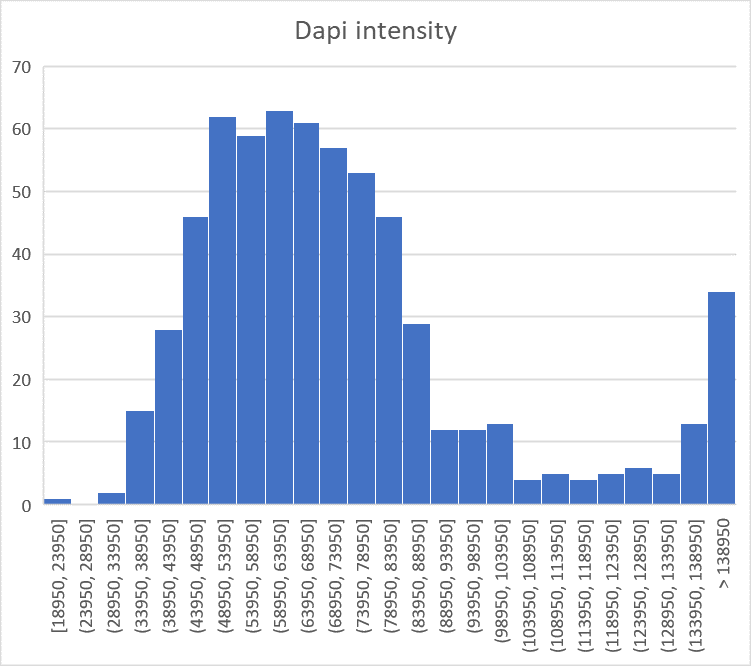


Supplementary figure 6: Cumulative population doublings for included MSC lines


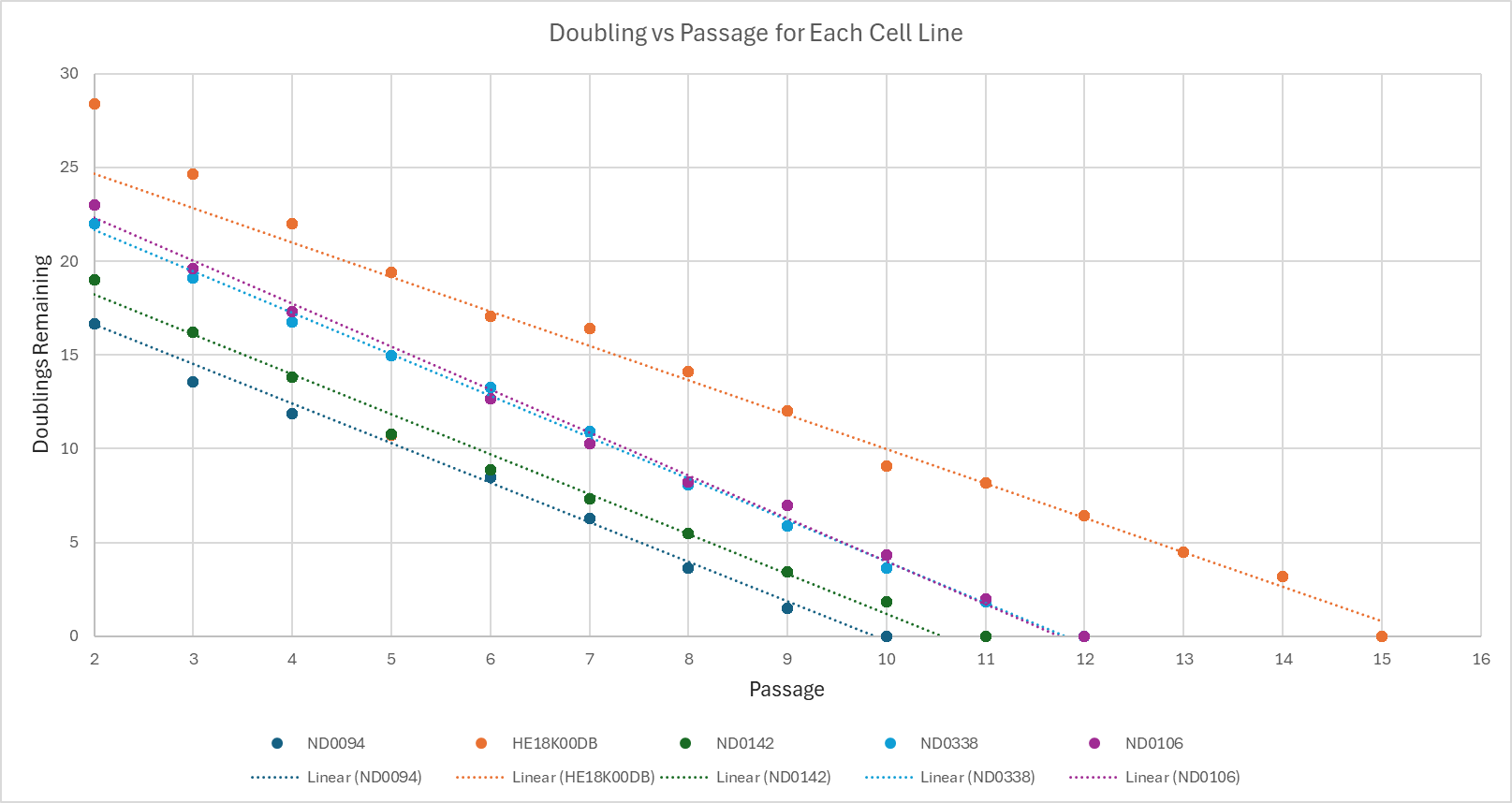

Supplement: Supplementary file 1 — Supplementary file1 (DOCX 1.34 MB) [file 12015_2024_10778_MOESM1_ESM.docx]
